# A Methodology for Determining Which Diseases Warrant Care in a High-Level Containment Care Unit †

**DOI:** 10.3390/v11090773

**Published:** 2019-08-22

**Authors:** Theodore J. Cieslak, Jocelyn J. Herstein, Mark G. Kortepeter, Angela L. Hewlett

**Affiliations:** 1 Department of Epidemiology, College of Public Health, University of Nebraska Medical Center, Omaha, NE 68198, USA; 2 Department of Environmental, Agricultural & Occupational Health, College of Public Health, University of Nebraska Medical Center, Omaha, NE 68198, USA; 3 Department of Medicine, Division of Infectious Diseases, College of Medicine, University of Nebraska Medical Center, Omaha, NE 68198, USA

**Keywords:** high-level containment care, biocontainment, highly hazardous communicable disease, Ebola virus disease, infectivity, communicability

## Abstract

Although the concept of high-level containment care (HLCC or ‘biocontainment’), dates back to 1969, the 2014–2016 outbreak of Ebola virus disease (EVD) brought with it a renewed emphasis on the use of specialized HLCC units in the care of patients with EVD. Employment of these units in the United States and Western Europe resulted in a significant decrease in mortality compared to traditional management in field settings. Moreover, this employment appeared to significantly lessen the risk of nosocomial transmission of disease; no secondary cases occurred among healthcare workers in these units. While many now accept the wisdom of utilizing HLCC units and principles in the management of EVD (and, presumably, of other transmissible and highly hazardous viral hemorrhagic fevers, such as those caused by Marburg and Lassa viruses), no consensus exists regarding additional diseases that might warrant HLCC. We propose here a construct designed to make such determinations for existing and newly discovered diseases. The construct examines infectivity (as measured by the infectious dose needed to infect 50% of a given population (ID_50_)), communicability (as measured by the reproductive number (R_0_)), and hazard (as measured by morbidity and mortality). Diseases fulfilling all three criteria (i.e., those that are highly infectious, communicable, and highly hazardous) are considered candidates for HLCC management if they also meet a fourth criterion, namely that they lack effective and available licensed countermeasures.

## 1. Introduction

High-level containment care (HLCC, also known as ‘biocontainment’) units are highly specialized patient care facilities that employ a number of engineering and administrative controls exceeding those found in more conventional infection control patient care environments (such as airborne infection isolation rooms (AIIRs)). HLCC facilities typically have design features that include designated “cold, warm and hot” or “clean and contaminated” zones; negative pressure and specialized air handling; autoclaves for waste management; video monitoring systems; and secured access. Other assets of HLCC units include trained teams of healthcare workers, a set of practiced protocols designed to minimize risk of nosocomial pathogen spread, ready access to laboratory testing (often as a point-of-care laboratory located in the unit) and critical care modalities. Prior to the 2014–2016 outbreak of Ebola virus disease (EVD) in West Africa, only four units with a total capacity of fewer than 20 beds existed within the United States. Although a few dozen medical centers have since developed some HLCC capability, HLCC doctrine remains in its infancy. Smith and colleagues [1] published initial guidance in 2006 on the construction and operation of HLCC units; however, no widely accepted consensus exists regarding the specific components of HLCC, or the diseases that warrant such care.

During the 2014–2016 West African outbreak, the mortality rate among the 28,652 suspected, probable, and confirmed victims was 40% [2]. That rate fell to 18.5% among expatriate patients cared for in US and European HLCC units. Perhaps equally important, no secondary cases or evidence of nosocomial transmission occurred within these units. While these seeming improvements are based upon a very small number of patients (27 persons were managed in HLCC units in the US and Europe during that outbreak), it lends support to the notion that such units, or at least the provision of critical care and enhanced medical capabilities provided within these units compared with what was available in the field environment, may prove beneficial in EVD case management and in reducing the risk of nosocomial transmission [3]. The impact of the investigational products that were given to many of the patients cannot be ascertained, but the ability to provide these products and follow applicable FDA regulations for human subjects research is another advantage of HLCC. On the other hand, HLCC care is extraordinarily expensive and the number of HLCC beds is likely to remain extremely limited, making it unlikely that such care (or at least the engineering controls employed in the delivery of such care) can be provided with the same degree of robustness in developing nations and austere environments.

In addition to EVD, a very small number of patients with other viral hemorrhagic fevers (VHFs), such as Marburg, Lassa, and Crimean-Congo hemorrhagic fever (CCHF) have also been successfully cared for in such facilities. Moreover, China developed an entire specialized containment facility for managing patients with Severe Acute Respiratory Syndrome (SARS) during the 2003 outbreak of that disease [4], and multiple other facilities have managed patients infected with SARS and Middle East Respiratory Syndrome (MERS) under HLCC conditions. With these successes has come an increasing appreciation for the capabilities of such units and the prudence of considering their use in the management of patients with highly hazardous communicable diseases (HHCDs). Despite this appreciation, no formal consensus exists among HLCC unit leaders and policymakers regarding the specific pathogens and diseases that warrant the use of the very limited number of these resource-intensive units [5]. We propose here a construct for evaluating which pathogens, and the diseases they cause, may be suitable for management in HLCC units, and provide examples of pathogens that fit within this construct.

## 2. Background

Three properties of an infectious disease factor into our initial assessment: infectivity, communicability, and hazard. While many diseases are highly infectious, highly contagious, or highly hazardous, very few possess all three attributes. It is those few diseases that we argue warrant HLCC care when such care is accessible.

Infectivity is typically expressed in terms of the ‘Infectious Dose 50′ or ID_50_, the dose (cells or colony-forming units in the case of bacteria; virions or plaque-forming units in the case of viruses) necessary to infect, on average, 50% of exposed individuals. Some pathogens may be highly infectious, but lack a propensity for person-to-person spread. For example, *Coxiella burnetii*, the causative agent of Q-fever, is among the most infectious pathogens known, with a single bacterial cell capable of causing human infection [6]. The disease is rarely transmitted from person-to-person, however, and thus poses little risk to contacts or health care personnel. Proper management of this disease (which also has a very low mortality rate) would not require an HLCC unit. 

A similar argument might be made with many other infectious diseases, including brucellosis, wherein the causative pathogens (Brucella sp) are included as Centers for Disease Control and Prevention (CDC) Category B agents of bioterrorism. Practically speaking, infectivity may be the least important of the three properties in determining the need for HLCC care. This derives from the fact that an exposure (via sneeze or cough) typically involves an inoculum order of magnitude greater than the ID_50_. Conversely, infectious dose is an important consideration when evaluating the efficacy of masks and respirators in preventing nosocomial transmission.

Communicability, or contagiousness, is typically expressed in terms of the reproductive number, or R_0_, where R_0_ = βcD (β denotes the probability of transmission assuming ‘effective’ contact, c denotes the rate of that contact, and D is the duration of infectivity). In simple terms, R_0_ denotes the number of secondary cases resulting from a single primary case in the absence of medical interventions (such as immunization). It can also be viewed as the ‘epidemic threshold’, with agents having an R_0_ > 1 possessing the capability to cause an outbreak. The greater the R_0_ value, the more likely that an outbreak could occur. While pathogens with an R_0_ less than one but greater than zero are not likely to lead to large outbreaks, they nonetheless pose a risk of isolated and nosocomial transmission. Mumps provides an example of a disease that is quite contagious, with an R_0_ value of 3–10 [7] but that is not particularly hazardous and not as infectious as many other respiratory pathogens (while ID_50_ data for Mumps is scant, the ID_50_ is likely significantly higher than it is for diseases such as measles and influenza). Therefore, mumps would not require HLCC care (the existence of an effective vaccine further obviates this need). Nor would norovirus, which is highly communicable, but not particularly hazardous and perhaps slightly less infectious than some pathogens, with an ID_50_ of ~6430 viral particles (when assessed in gnotobiotic pigs) [8].

Hazard is usually expressed in terms of morbidity and mortality. Inhalational anthrax has a mortality rate that approaches 100% in the absence of prompt antimicrobial therapy, yet it is not particularly infectious, with an ID_50_ estimated at 8000–40,000 spores [9]. Moreover, as it primarily affects mediastinal lymph nodes rather than lung parenchyma, it is not transmissible from person-to-person. Caregivers of anthrax patients are at no risk of nosocomial acquisition of disease and HLCC care is thus unnecessary. The same would hold true of botulism, an intoxication caused by toxin exposure rather than a true infection with *Clostridium botulinum*; as such it is neither infectious nor contagious, but it is highly hazardous [10].

Certain diseases fulfill two of the above criteria. *Francisella tularensis* spp. *tularensis*, the causative agent of type A tularemia, a highly hazardous disease with a mortality rate as high as 30–60% for the pneumonic form of the disease in the pre-antibiotic era [11], is included among the CDC’s Category A agents of bioterrorism. Type A tularemia has an ID_50_ of approximately 10 organisms [12], yet tularemia is not transmissible from person-to-person and would therefore not warrant care in an HLCC unit.

**Figure 1:**
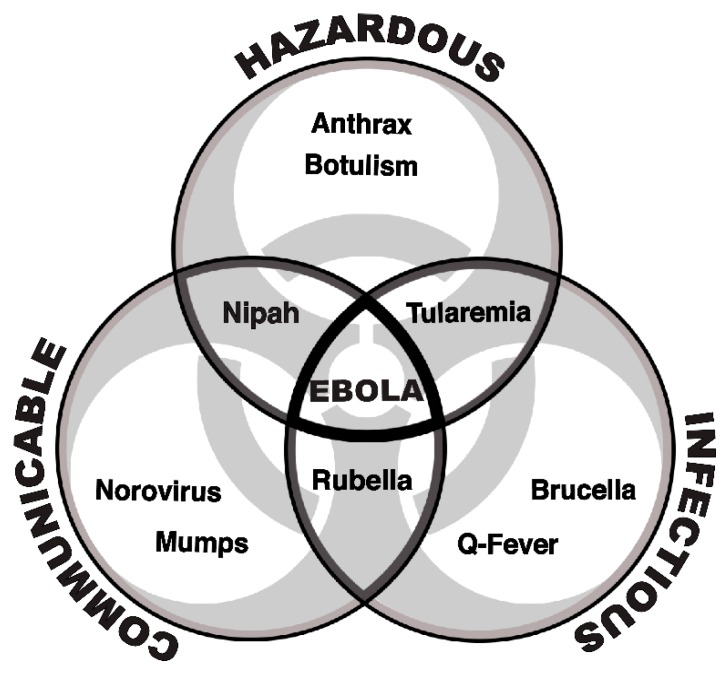
Construct used in determining diseases warranting care in a high-level containment care unit.

The three aforementioned criteria of infectivity, communicability, and hazard are portrayed graphically in Figure 1. It is where these criteria intersect that we determine the need for HLCC, although such evaluation ultimately involves consideration of a fourth criterion, namely the availability of licensed, effective medical countermeasures. 

One might argue that measles fulfills all three of the previously described criteria. It is among the most communicable diseases known, with an R_0_ of 12–18, and is highly infectious. Additionally, having caused an estimated 545,000 annual deaths worldwide as recently as 2000 [13], it is also highly hazardous. However, a very effective vaccine against measles protects healthcare workers from nosocomial transmission. Measles patients requiring inpatient care thus would not require HLCC, but they would require airborne precautions including the use of an AIIR. The situation is similar for polio; a highly infectious, highly contagious, and quite hazardous disease. Polio, however, nears its global eradication thanks to the existence of very effective vaccines. Patients with polio would thus not require HLCC care, as the fear of nosocomial spread should be alleviated by caregiver immunization. 

By applying this fourth criterion to the short list of diseases fulfilling the first three criteria, we further trim the list of diseases for which HLCC care might be warranted. We previously published a proposed list of diseases potentially warranting HLCC care [14]. This list is not necessarily comprehensive and was based, in part, on an informal survey taken of HLCC unit leaders in the United States. However, the list is primarily intended to provide a framework for considering which diseases warrant consideration for care in an HLCC unit; the authors are certainly open to consideration of other diseases or newly emerging diseases that might be added to the list in the future. In this paper, we expand upon the previous work and present R_0_ and ID_50_ data for those diseases based on a thorough review of the relevant literature (Table 1).

## 3. Methods

A literature search was performed in PubMed/MEDLINE (January 1966 through May 2019) using the terms (“reproductive number” OR “infectious dose” OR “infectivity”) coupled with each of the diseases listed in the table (e.g., (“reproductive number” OR “infectious dose” OR “infectivity”) AND “Ebola”). A separate search was conducted for each disease, and titles and abstracts were screened for relevance. Results of this search are presented in the table.

## 4. Discussion

High-level containment care units offer the advantage of robust engineering controls in conjunction with established and exercised protocols implemented by highly trained teams of healthcare workers. These enable provision of the highest level of patient care in a challenging environment, with utmost attention paid to infection control in order to protect healthcare workers and the community at large from HHCDs. The list of pathogens that fulfill the criteria of infectivity, communicability, and hazard is relatively short. We argue that patients infected with these uncommon diseases warrant HLCC care, when such care is available.

The list of diseases we propose was first generated in 2017 from an informal survey of HLCC leaders in the United States who were asked to select, from among a list of potential infections, those infections that should be considered candidates for HLCC care. Therefore, we recognize that the list is not all-inclusive and that additional pathogens might be proposed by others. We have not applied strict numeric cut-offs for the attributes already mentioned (infectious, hazardous, communicable), as we believe that each pathogen is best considered based on its properties as a whole, with the attributes having differing degrees of relative importance. Several diseases discussed in this paper have R_0_ values less than one, but greater than zero. Among these are Nipah, Monkeypox, Crimean-Congo Hemorrhagic Fever (CCHF), and the New World Arenaviral hemorrhagic fevers (Junin, Machupo, Sabia). While these diseases are unlikely to lead to epidemics, the risk of limited transmission, especially in the nosocomial setting where caregivers might experience intense exposure, is high. For this reason, we maintain that such diseases also warrant HLCC.

HLCC units might similarly be utilized for patients infected with new or emerging pathogens where characteristics such as transmissibility are not well established. SARS, MERS, and the various viral hemorrhagic fevers were all included in this category when they first surfaced. Although patients infected with HHCDs have been successfully managed in conventional settings when HLCC is unavailable, doing so is extremely challenging and may increase the risk of nosocomial spread of the disease if strict adherence to infection control principles is not maintained. Therefore, we advocate that patients infected with the pathogens in the table be managed in HLCC units, whenever possible. 

We also realize that new diseases will continue to emerge and that some of these will present a risk of contagion and nosocomial spread. We thus advocate that these diseases be evaluated as potential candidates for HLCC based on the criteria we have proposed. We further understand that, although fundamental properties of the agents may not change, the development of effective countermeasures might cause us to reconsider whether they should be retained on our list. For example, a significant amount of data is being generated in the current Ebola outbreak in the Democratic Republic of the Congo on vaccines and therapeutics against EVD. If any of these products should become licensed, a re-evaluation would be warranted, although one might argue that the vaccine or therapeutics would have to be highly effective, because the consequences of Ebola infection are so great. In that vein, we have included smallpox (Variola virus) on our list, despite having an effective vaccine available, as we would argue that in the current setting, the public health consequences of a smallpox outbreak are so considerable that it would reasonably be another candidate for HLCC. Similarly, although monkeypox does not appear to spread as efficiently, nor is it as deadly as smallpox, we would advocate for placing a suspected monkeypox patient into HLCC until such time as they are ruled out for smallpox. 

The number and capabilities of HLCC units throughout the world have increased in recent years; nonetheless these assets remain a relative rarity due to the significant cost and resources needed to build and maintain them. We advocate that funding for biopreparedness efforts, including outbreak investigation and response efforts; building of infrastructure; development of novel diagnostics, therapeutics and vaccines; and maintenance of this preparedness against HHCDs remain a high priority. We believe that this is critical if we are to effectively investigate, clinically manage, and ultimately prevent the transmission of HHCDs.

## 5. Conclusions 

As the science of infection control advances, the emphasis on HLCC over the past 10 years has increased, both in the United States and internationally. In parallel with this emphasis and awareness, the number and capabilities of HLCC (or ‘biocontainment’) units has also increased. Despite this, there is thus far no agreed upon list of the diseases that should be cared for in an HLCC setting. We are not advocating for such a requirement; however, we have proposed a list of diseases and a methodology for determining those diseases that we believe should be considered candidates for care in an HLCC environment, when such a facility is available. Because better characterization of diseases will occur over time, and effective licensed therapeutics and vaccines will be developed, we expect this list will evolve over time. We hope that having an assessment tool such as we have proposed will help clinicians and administrators determine the optimum environment in which to care for their patients to maximize benefit to the patient while minimizing the risk of transmission to others in the nosocomial environment and, ultimately, in the community.

## Figures and Tables

**Table 1:** ID_50_ and R_0_ data for putative HLCC pathogens.

Pathogen	Mechanism of PTP Spread	ID_50_	R_0_	References
Ebola	Blood & Body Fluids	1-10 aerosolized organisms	1.3–2.53	[15,16,17,18,19,20,21,22]
Marburg	Blood & Body Fluids	1-10 aerosolized organisms	1.59	[11,23]
Lassa	Blood & Body Fluids	1-10 aerosolized organisms	1.23–1.33	[11,24]
Lujo	Scant data; Presumably Blood & Body Fluids	No data	No data	
Junin	Blood & Body Fluids	No data	<1	[25,26]
Machupo	Blood & Body Fluids	No data	<1	[21,22]
Guanarito	Scant data; Presumably Blood & Body Fluids	No data	No data	
Sabia	No data	No data	<1	[21]
CCHF	Blood & Body Fluids	No data	<1	[21,27]
SARS	Respiratory Droplets; Possibly Droplet Nuclei	No data	2.2–3.6	[28,29,30,31]
MERS	Respiratory Droplets; Possibly Droplet Nuclei	No data	0.60–11.5 ^1^	[32,33,34,35,36]
H5N1 Influenza	Respiratory Droplets; Possibly Droplet Nuclei	1000 viral particles ^2^	1.14	[37]
H7N9 Influenza	Respiratory Droplets; Possibly Droplet Nuclei	1000 viral particles	0.1–0.47	[37]
Smallpox	Droplet Nuclei, Scabs	1–100	3.5–7.0	[38,39,40,41]
Monkeypox	Respiratory Droplets; Possibly Droplet Nuclei and Scabs	No data	0.32	[42]
Nipah	Respiratory Droplets	No data	0.33	[43]
Hendra	No Data	No data	No data	
Pneumonic Plague	Respiratory Droplets	100 to 500 organisms by inhalation	1.3–3.5	[44,45,46,47,48]
XDR-TB	Droplet Nuclei	<10 bacilli ^3^	1.97	[49,50]

^1^ Estimates from South Korean and Saudi studies vary widely; ^2^ Influenza data is not specific to these strains; ^3^ TB data is not specific to XDR strains. Key: HLCC = High Level Containment Care; PTP = Person-to-Person; CCHF = Crimean-Congo Hemorrhagic Fever; SARS = Severe Acute Respiratory Syndrome; MERS = Middle East Respiratory Syndrome; XDR-TB = Extensively Drug-Resistant Tuberculosis.
